# A Compact Three-Layer Stacked Feed Network Integrating a Quad-Ridged Orthomode Transducer and Diplexers for Dual-Band Millimeter-Wave Applications

**DOI:** 10.3390/mi17060752

**Published:** 2026-06-21

**Authors:** Yuanjun Shen, Tianling Zhang, Jiayin Guo, Pengpeng Chu

**Affiliations:** 1 National Key Laboratory of Radar Detection and Sensing, Xidian University, Xi’an 710071, China; yuanjun.shen@xidian.edu.cn; 2 State Key Laboratory of Mobile Network and Mobile Multimedia Technology, Department of RCH System, ZTE Corporation, Xi’an 710000, China; guo.jiayin@zte.com.cn (J.G.); chu.pengpeng1@zte.com.cn (P.C.)

**Keywords:** dual-band feed network, orthomode transducer (OMT), quad-ridged waveguide, waveguide diplexer, stepped-impedance filter, millimeter-wave

## Abstract

A compact, low-profile dual-band feed network operating at 37–40 GHz (Ka-band) and 70–86 GHz (E-band) is presented for millimeter-wave backhaul applications. The proposed network integrates a quad-ridged orthomode transducer (OMT) and four ridge-waveguide diplexers into a three-layer all-metal stacked architecture, eliminating the cascaded inter-stage flanges of conventional feed chains and yielding a monolithic-like assembly that is mechanically robust and CNC-friendly for mass production. Stepped-impedance matching stubs in the OMT junction provide broadband matching across the widely separated bands, while compact ridge-waveguide T-junction diplexers, comprising stepped-impedance low-pass filters and rectangular high-pass paths, perform the spectral separation. Back-to-back measurements of the fabricated prototype demonstrate an insertion loss below 0.6 dB across both bands. The measured VSWR at the four output ports remains below 1.5 across both bands, and the port-to-port isolation exceeds 32 dB at the Ka-band and 45 dB at the E-band. The proposed network thus offers a highly integrated, low-loss solution for next-generation dual-band mmWave links.

## 1. Introduction

With the rapid growth of wireless data traffic and the deployment of 5G/6G backhaul and satellite broadband services, millimeter-wave (mmWave) technology has become a key enabler for future high-capacity networks owing to its large available bandwidth and low latency [1,2]. Among the various mmWave bands, the licensed 70/80 GHz E-band has gained particular interest for fiber-like point-to-point backhaul links, with several recent studies reporting compact E-band terminals and beam-steerable front-ends [3]. To exploit multiple mmWave bands within a single radio-frequency front-end, dual-band feed networks capable of simultaneously handling widely separated frequencies, such as 38 GHz and 80 GHz, are increasingly demanded for compact backhaul terminals, beam-steerable antenna front-ends, and shared-aperture reflector systems [4,5,6].

Designing such dual-band feed networks, however, presents significant technical challenges. A primary bottleneck is realizing a compact, low-profile architecture supporting widely separated bands while simultaneously maintaining low insertion loss, dual orthogonal polarizations, high inter-band isolation, and precise mechanical alignment. Existing solutions generally rely either on cascaded feed chains assembled from discrete OMT, transition, and filter modules, or on frequency-selective surface (FSS)-based architectures [7,8,9]. Cascaded chains suffer from bulky longitudinal dimensions, multiple inter-stage flanges, and accumulated assembly tolerances, whereas FSS-based approaches introduce optical complexity and potential high-frequency losses. Furthermore, in such wideband integrated feeds, insufficient suppression of higher-order modes can cause severe inter-modulation interference, thereby degrading spectral purity [10]. Hence, a highly integrated feed architecture combining structural simplicity with rigorous mode control is urgently required.

In recent years, quad-ridged waveguide structures have emerged as promising candidates for broadband polarimetric applications, offering wide impedance bandwidth and excellent polarization purity [11,12,13]. Quad-ridged flared horns (QRFHs) have been successfully employed in radio telescopes to achieve decade-bandwidth performance [14,15]. In parallel, the turnstile-junction OMT topology has been extensively investigated and refined for broadband, polarization-pure operation, with reported designs ranging from full-band rectangular-waveguide implementations to ultra-thin, octave-bandwidth, and mmWave variants [16,17,18,19,20]. On the multiplexing side, ridge-based waveguide diplexers and multiplexers have been developed to efficiently separate frequency channels within a compact footprint [21,22,23]. Nevertheless, the integration of these disparate components, specifically the quad-ridged OMT, transitions, and diplexers, into a single low-profile module for mmWave backhaul remains a rarely explored area. The main difficulty involves balancing fabrication tolerances against stringent filtering requirements within a confined geometry, especially at E-band frequencies where micron-level alignment is critical [24].

**Figure 1:**
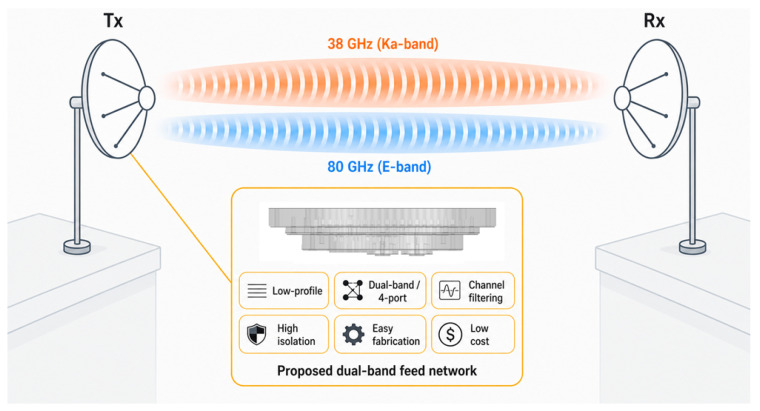
Application scenario and key attributes of the proposed dual-band feed network.

To address these limitations, this paper proposes a low-profile dual-band feed network based on a three-layer all-metal stacked topology, targeted at 38/80 GHz point-to-point millimeter-wave backhaul links of the kind illustrated in Figure 1. As highlighted in the figure, the proposed module is designed to deliver, within a single compact assembly, the key attributes required by such links: a low axial profile, simultaneous dual-band operation with four polarization-pure output ports, built-in channel filtering, high inter-port isolation, and an all-metal CNC-friendly construction that supports low-cost mass production. To realize these features, the design integrates a quad-ridged OMT and four ridge-waveguide diplexers into a single monolithic-like block, eliminating the inter-stage flanges of conventional cascaded chains. Back-to-back measurements demonstrate an insertion loss below 0.6 dB across both 38 GHz and 80 GHz. Furthermore, the measured VSWR at the four output ports remains below 1.5 across both bands and the inter-port isolation exceeds 32 dB at Ka-band and 45 dB at E-band, confirming broadband matching and high-isolation operation. The remainder of this paper is organized as follows. Section 2 describes the design methodology, including the network architecture, the quad-ridged OMT, and the diplexing network. Section 3 presents the fabrication and measurement results. Finally, Section 4 concludes the paper.

## 2. Design of the Dual-Band Feed Network

### 2.1. Feed Network Architecture

**Figure 2:**
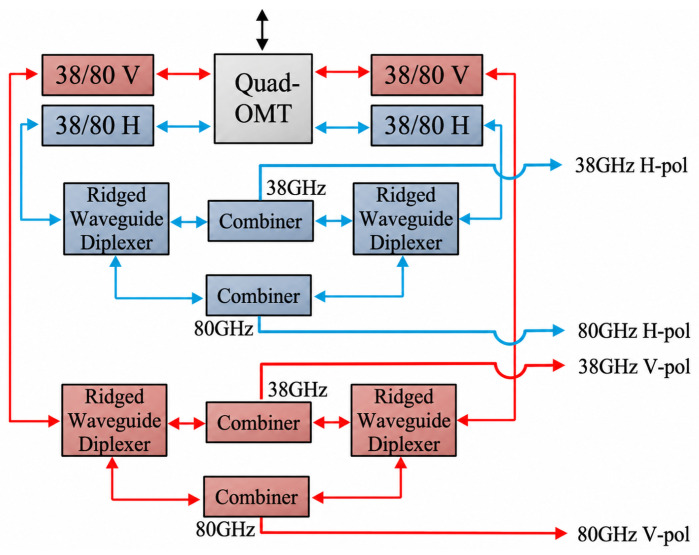
Signal flow block diagram of the proposed dual-band feed network. The red and blue paths indicate the V-polarized and H-polarized signal channels, respectively.

The proposed dual-band feed network is designed to handle simultaneous operation at 38 GHz (Ka-band) and 80 GHz (E-band) with dual orthogonal linear polarizations. Its signal flow follows the topology illustrated in Figure 2. Signals enter through the common quad-ridged waveguide port and propagate directly into the OMT, which separates the two orthogonal polarization modes. Each polarization path is subsequently routed to a pair of ridge-waveguide diplexers that spectrally separate the 38 GHz and 80 GHz components, yielding four independent output ports: horizontal (H) and vertical (V) polarization for each band. In this work, the target operating passbands are defined as 37 to 40 GHz for the Ka-band, and 70 to 86 GHz for the E-band. Therefore, the terms “38 GHz band” and “80 GHz band” refer to finite operating passbands centered around 38 GHz and 80 GHz, rather than two discrete frequency points.

The proposed feed network is intended to serve as the backend feeding module of a standard dual-reflector antenna system. In the complete antenna configuration, the feed network can be mounted at the back of the reflector antenna assembly. The common quad-ridged waveguide port is connected to the circular-waveguide/dual-mode horn feed through a compact quad-ridged-to-circular waveguide transition. The dual-mode horn then illuminates the sub-reflector, and the reflected field is further collimated by the main reflector. Therefore, the proposed network provides four separated backend channels for the Ka-band H/V and E-band H/V signals, while maintaining a single, shared aperture-side interface toward the dual-reflector radiating structure.

**Figure 3:**
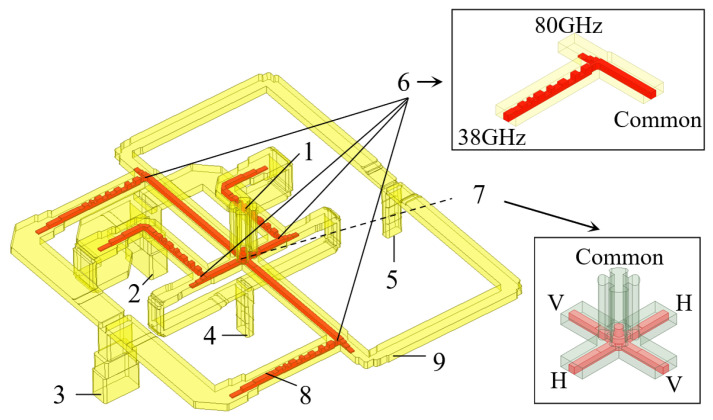
Perspective view of the internal waveguide network of the proposed feed system. (1: common quad-ridged waveguide port; 2–3: 38 GHz H/V-pol ports; 4–5: 80 GHz H/V-pol ports; 6: ridge-waveguide diplexer; 7: OMT junction; 8: stepped-impedance ridge filter; 9: rectangular waveguide.)

The electromagnetic functionality of the feed network is defined by the complete internal waveguide topology depicted in Figure 3. This vacuum-core model reveals the routing of signals from the common port to the separated backend ports. The signal path begins at the central common quad-ridged waveguide port (component 1) and enters the turnstile OMT junction (component 7), where it is distributed into four symmetrical arms. In each arm, a ridge-waveguide diplexer (component 6), with an integrated stepped-impedance filter (component 8) and a rectangular waveguide section (component 9), separates the two frequency bands. The outputs are recombined to synthesize the final H- and V-polarization ports for both 38 GHz (components 2–3) and 80 GHz (components 4–5).

### 2.2. Wideband Quad-Ridged OMT

**Figure 4:**
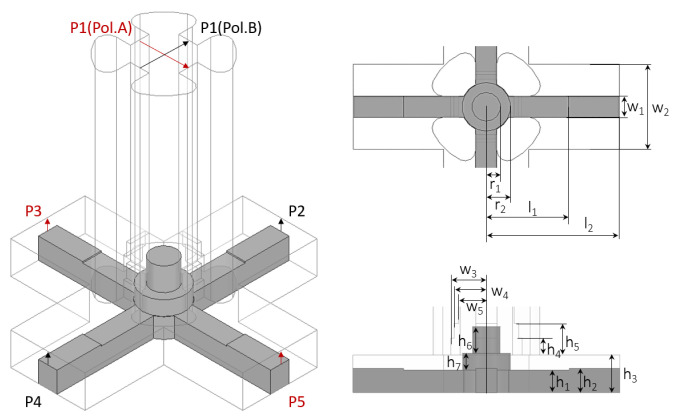
Geometry of the quad-ridged OMT with annotated design parameters. The colored labels are used to distinguish the ports associated with the two orthogonal polarization channels. P1 denotes the common quad-ridged waveguide port, where Pol. A and Pol. B represent the two orthogonal polarizations, while P2 to P5 denote the four rectangular-waveguide ports.

The core of the feed network is the wideband quad-ridged OMT, corresponding to the central region of the waveguide network labeled as components 1 and 7 in Figure 3. The common quad-ridged waveguide port (1) interfaces directly with the OMT turnstile junction (7), supporting two orthogonal dominant quad-ridged guided modes that correspond to the H- and V-polarizations across both operating bands. The geometry of the OMT with annotated design parameters is shown in Figure 4, and the corresponding dimensions are listed in Table 1.

To achieve dual-band operation covering the widely separated Ka-band and E-band, the impedance transformation within the OMT junction is critical. A standard uniform ridge profile fails to provide sufficient matching bandwidth for both frequency ranges simultaneously. Therefore, stepped metallic matching stubs are embedded in the central OMT junction (7). These stubs feature a multi-section stepped impedance profile optimized to suppress higher-order modes and minimize reflections at the discontinuity between the common quad-ridged port and the branching arms.

**Figure 5:**
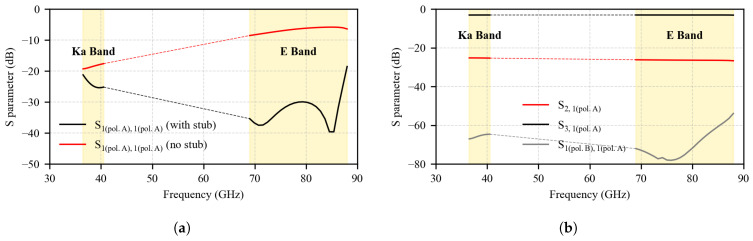
Simulated scattering parameters of the quad-ridged OMT junction for one polarization mode: (**a**) $S_{1(\mathrm{pol}.A),1(\mathrm{pol}.A)}$ with and without matching stubs, and (**b**) $S_{2,1(\mathrm{pol}.A)}$, $S_{3,1(\mathrm{pol}.A)}$, and $S_{1(\mathrm{pol}.B),1(\mathrm{pol}.A)}$. The dashed straight segments simply connect the separated solid curve sections across the out-of-interest frequency ranges and are not used for performance evaluation.

To validate the effectiveness of this stepped design, the simulated scattering parameters of the OMT are presented in Figure 5. Figure 5a compares the reflection coefficient ($|S_{11}|$) at the common port with and without the optimized matching stubs. As observed, the proposed stepped structure significantly improves the impedance matching, achieving $|S_{11}|$ better than −20 dB across both the 38 GHz and 80 GHz bands for both orthogonal polarization modes. Figure 5b presents the transmission coefficients to the two output arms ($S_{2,1(\mathrm{pol}.A)}$ and $S_{3,1(\mathrm{pol}.A)}$) along with the cross-polarization coupling ($S_{1(\mathrm{pol}.B),1(\mathrm{pol}.A)}$). The results confirm that signals are efficiently routed to the designated polarization arms with cross-polarization leakage suppressed below −40 dB across both bands. This ensures consistent amplitude and phase response, which is vital for the subsequent polarization synthesis and diplexing operations.

**Figure 6:**
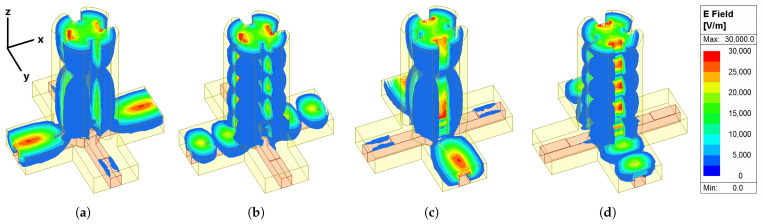
Simulated E-field distribution in the OMT structure when feeding at the common port: (**a**) 40 GHz under x-polarization, (**b**) 80 GHz under x-polarization, (**c**) 40 GHz under y-polarization, and (**d**) 80 GHz under y-polarization.

To further illustrate the polarization separation mechanism, the simulated electric-field distributions within the OMT structure are presented in Figure 6. When the common port is excited with x-polarization at 40 GHz (Figure 6a), the field energy is predominantly directed to the corresponding horizontal arm, while at 80 GHz (Figure 6b), the field propagates through the same arm with minimal leakage to the orthogonal channel. An analogous behavior is observed under y-polarization excitation, as shown in Figure 6c,d. These field patterns confirm the effective broadband polarization separation capability of the quad-ridged OMT across both frequency bands.

### 2.3. Integrated Diplexing and Routing Network

Following the polarization separation stage, the signals propagate into the diplexing and routing network. To maintain a compact footprint while supporting wideband operation, ridge waveguide technology is employed throughout the routing network. Each of the four OMT arms connects to a compact ridge-waveguide T-junction diplexer, labeled as component 6 in Figure 3, which serves as the spectral branching point for the 38 GHz and 80 GHz bands.

**Figure 7:**
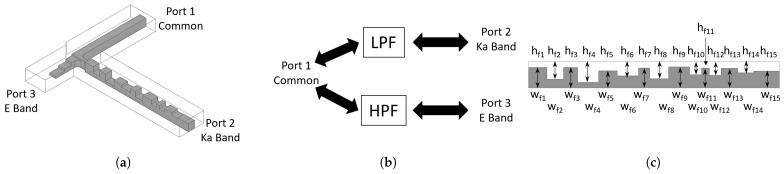
Structure of the ridge-waveguide diplexer unit: (**a**) 3D model showing the T-junction with low-pass and high-pass paths, (**b**) equivalent functional schematic indicating the LPF and HPF branches, and (**c**) detailed geometry of the stepped-impedance ridge filter with annotated dimensions.

The structure of the individual diplexer unit is illustrated in Figure 7. As shown in the 3D model (Figure 7a) and the equivalent schematic (Figure 7b), the diplexer adopts a hybrid filtering topology with two distinct frequency-selective paths. The low-frequency path for the 38 GHz band incorporates a stepped-impedance low-pass filter (component 8), whose detailed ridge profile is shown in Figure 7c. This filter is constructed using a series of capacitive ridge steps. By properly varying the ridge height, a cascade of impedance discontinuities is introduced, where each ridge step behaves predominantly as a capacitive loading due to the strong electric-field concentration in the ridge gap. Together with the intervening waveguide sections, these discontinuities form a stepped-impedance low-pass response. As a result, the 38 GHz signal experiences a smooth impedance transition with low insertion loss, whereas the 80 GHz signal is strongly reflected and attenuated. The dimensions of the 15-section stepped filter are given in Table 2.

Conversely, the high-frequency path for the E-band utilizes a rectangular waveguide section, as denoted by component 9 in Figure 3. This path adopts a standard WR-12 rectangular waveguide cross section, with a broad-wall dimension of $a_{\mathrm{HP}}=3.10\;\mathrm{mm}$ and a narrow-wall dimension of $b_{\mathrm{HP}}=1.55\;\mathrm{mm}$. The cutoff frequency of the dominant ${\mathrm{TE}}_{10}$ mode can be calculated as

(1) $$f_{c,\mathrm{TE}10}=\frac{c}{2a_{\mathrm{HP}}}\approx 48.4\;\mathrm{GHz},$$

where *c* is the speed of light in free space. This cutoff frequency is higher than the Ka-band operating passband but lower than the E-band operating passband. Therefore, the rectangular waveguide path behaves as an intrinsic high-pass section: the Ka-band signal is below cutoff and is naturally suppressed, whereas the E-band signal can propagate with low loss. In addition, the cutoff frequencies of the next higher-order modes, including ${\mathrm{TE}}_{20}$ and ${\mathrm{TE}}_{01}$, are approximately

(2) $$f_{c,\mathrm{TE}20}=\frac{c}{a_{\mathrm{HP}}}\approx 96.8\;\mathrm{GHz},\;f_{c,\mathrm{TE}01}=\frac{c}{2b_{\mathrm{HP}}}\approx 96.8\;\mathrm{GHz}.$$

These higher-order-mode cutoff frequencies are above the E-band operating range, which helps maintain single-mode propagation in the intended E-band passband.

**Figure 8:**
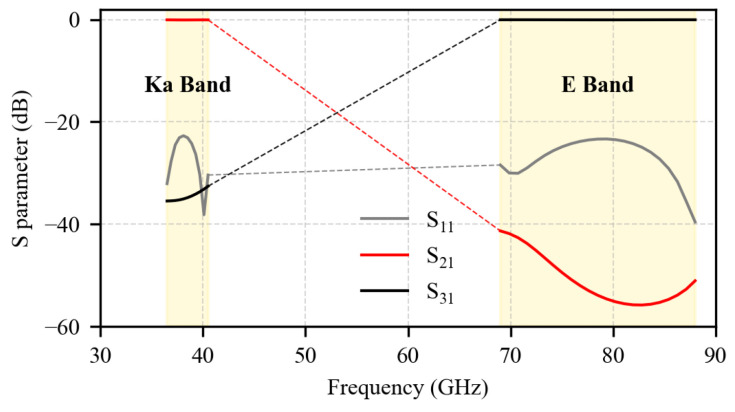
Simulated S-parameters of the individual ridge-waveguide diplexer unit showing the reflection ($S_{11}$) and transmission ($S_{21}$, $S_{31}$) coefficients. The dashed straight segments simply connect the separated solid curve sections across the out-of-interest frequency ranges and are not used for performance evaluation.

To quantify the performance of this critical component, the simulated scattering parameters of the individual diplexer unit are plotted in Figure 8. The results demonstrate excellent multiplexing characteristics: the transmission coefficients ($S_{21}$ and $S_{31}$) indicate an insertion loss of less than 0.3 dB in both passbands, out-of-band rejection exceeding 50 dB, and a reflection coefficient ($S_{11}$) at the common port below −20 dB across the operating bandwidths. These results validate the efficacy of the stepped-impedance design for achieving wideband diplexing performance.

**Figure 9:**
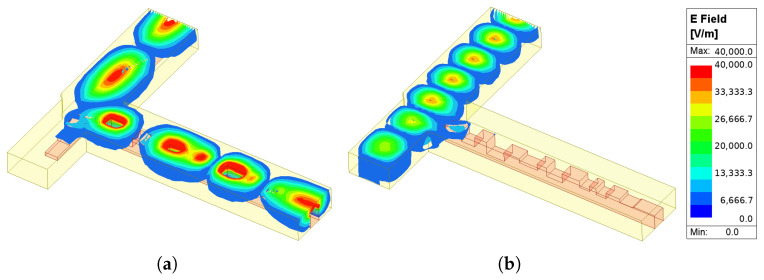
Simulated E-field distribution in the diplexer at (**a**) 40 GHz and (**b**) 80 GHz, illustrating the frequency-selective routing behavior.

**Figure 10:**
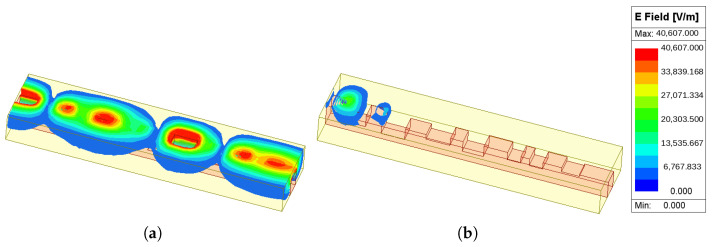
Simulated E-field distribution in the low-pass ridge filter arm at (**a**) 40 GHz, showing smooth transmission, and (**b**) 80 GHz, showing strong attenuation.

The frequency-selective behavior of the diplexer is further visualized through the E-field distributions at the two operating frequencies. As shown in Figure 9, at 40 GHz (Figure 9a), the field propagates through both the low-pass arm and the common section, while at 80 GHz (Figure 9b), the field is directed into the high-pass rectangular waveguide path with the low-pass arm effectively presenting a stopband. The field propagation within the low-pass arm alone is shown in Figure 10: at 40 GHz the field traverses the stepped-ridge section with smooth amplitude variation, confirming low insertion loss, while at 80 GHz, it is strongly attenuated along the ridge filter, demonstrating effective stopband rejection.

**Figure 11:**
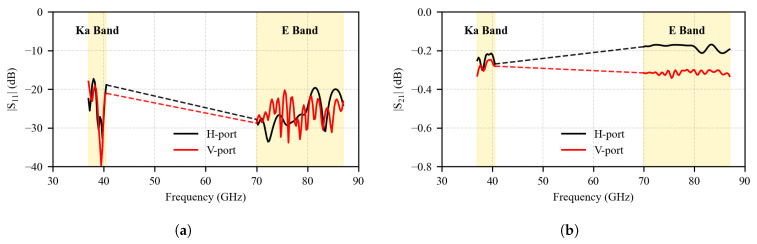
Simulated full-network S-parameter responses of the integrated dual-band feed network: (**a**) reflection coefficients and (**b**) transmission coefficients of the four ports. For clarity, the ports’ responses corresponding to the same polarization are grouped and plotted as H- and V-polarized curves over their respective operating bands. The dashed straight segments simply connect the separated solid curve sections across the out-of-interest frequency ranges and are not used for performance evaluation.

After the individual OMT and diplexer units have been verified, the full integrated feed network is further evaluated to confirm that the compact routing, cascading, and port recombination do not introduce additional mismatch or excessive loss. The simulated results are shown in Figure 11, where the four band/polarization channels, namely, the Ka-band H/V ports and the E-band H/V ports, are grouped by polarization for clarity. Specifically, the H-polarized curve represents the Ka-band H and E-band H responses within their respective passbands, while the V-polarized curve represents the corresponding Ka-band V and E-band V responses.

As shown in Figure 11a, the reflection coefficients remain below −17 dB for the Ka-band channels and below −20 dB for the E-band channels, indicating good impedance matching at all four output ports. The corresponding transmission coefficients in Figure 11b show an insertion loss of less than 0.35 dB across both operating bands. These full-network results demonstrate that the integrated OMT–diplexer architecture preserves the low-loss and broadband-matching characteristics observed in the individual component simulations, thereby providing a reliable basis for the subsequent fabrication and measurement validation.

## 3. Fabrication and Measurement

The proposed feed network is verified through fabrication and electrical characterization. First, the intrinsic insertion loss of a single feed unit is extracted using a back-to-back measurement at the common quad-ridged waveguide port. Subsequently, the input VSWR at each output port and the port-to-port isolation are measured to assess the impedance matching and the spectral/polarization separation of the network.

**Figure 12:**
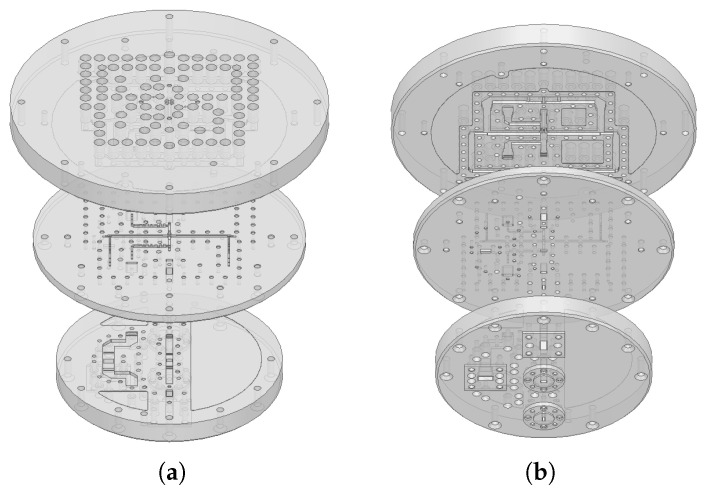
Exploded view of the three-layer all-metal stacked feed structure: (**a**) top view, and (**b**) bottom view.

The physical realization of the proposed feed network employs a stratified mechanical structure consisting of three aluminum layers, as shown in the exploded view of Figure 12. The stack is organized so that each layer accommodates a distinct functional region of the internal waveguide topology of Figure 3: the upper two layers host the common quad-ridged port and the turnstile OMT junction, while the bottom layer carries the four ridge-waveguide T-junction diplexers and the routing to the four output ports. The peripheral hole patterns visible in the top and bottom views (Figure 12a,b) correspond to the assembly screws and the precision alignment dowels that maintain registration between the layers. Unlike traditional cascaded feed chains that rely on multiple inter-stage flanges, this split-block architecture integrates the quad-ridged OMT and the waveguide diplexers into a single monolithic-like block, allowing the complex internal waveguide features to be machined with high fidelity using standard CNC milling tools while preserving mechanical robustness and simplified manufacturability.

**Figure 13:**
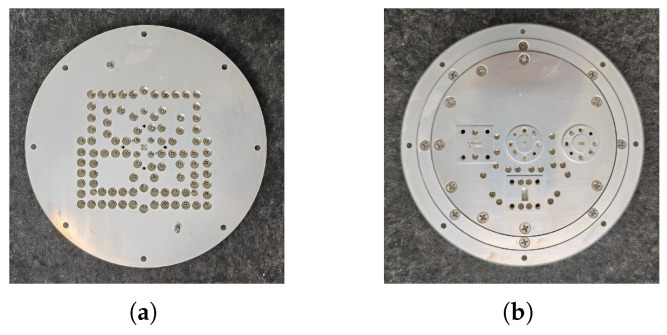
Photographs of the assembled three-layer feed network prototype: (**a**) top view showing the quad-ridged waveguide port, and (**b**) back view showing the four output ports.

Following this design, a prototype was fabricated by high-precision CNC milling from aluminum alloy (6061-T6) to ensure mechanical stability and high electrical conductivity. The complex internal features, including the OMT junction, matching stubs, and stepped-impedance filters, were machined with standard end-mill tools, and precision alignment dowel pins were used between the layers to maintain a registration tolerance within ±10 µm. The internal surfaces were polished to reduce conductor losses before the blocks were bolted together. The assembled prototype is shown in Figure 13: the top view (Figure 13a) reveals the quad-ridged waveguide port at the center, while the back view (Figure 13b) shows the four output ports corresponding to the H- and V-polarization channels of both bands.

**Figure 14:**
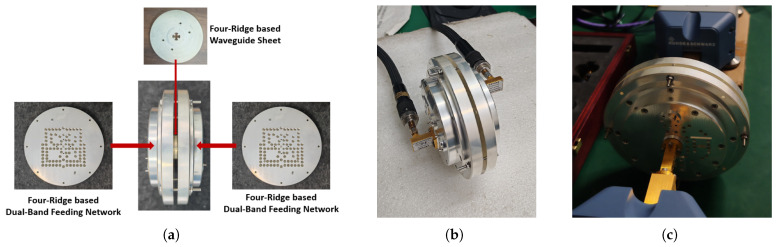
Back-to-back measurement setup: (**a**) two feed prototypes assembled at the common quad-ridged waveguide port, (**b**) measurement environment for Ka-band, and (**c**) measurement environment for E-band.

To extract the intrinsic insertion loss of the feed network without contributions from any radiating element, a back-to-back measurement was performed using two identical fabricated feed prototypes. As shown in Figure 14a, the two prototypes were assembled face-to-face at their common quad-ridged waveguide ports. The same screw holes and precision dowel-pin alignment features were used to ensure accurate registration between the two common ports. A thin metallic interface sheet was inserted at the contact plane to provide a continuous conducting interface and to minimize any additional air gap or discontinuity between the two quad-ridged waveguide openings.

Because the 38 GHz and 80 GHz bands fall outside the coverage of any single instrument available in the laboratory, the transmission coefficient ($S_{21}$) of the cascaded pair was measured in two stages using two complementary setups. The Ka-band response was characterized with an Agilent E8363B vector network analyzer (10 MHz–40 GHz), as shown in Figure 14b, while the E-band response was measured with a Rohde & Schwarz ZVA vector network analyzer extended by a ZVA-Z90 millimeter-wave frequency converter (60–90 GHz, WR-12 waveguide), which is shown in Figure 14c. The calibration reference planes were set at the external measurement ports, so the losses of cables, frequency converters, and external adapters were removed by calibration. The remaining measured transmission loss corresponds to two cascaded feed units and the common-port interface. Since the two feed prototypes are nominally identical and exhibit good impedance matching, the insertion loss of a single feed unit was extracted by halving the measured back-to-back transmission loss.

**Figure 15:**
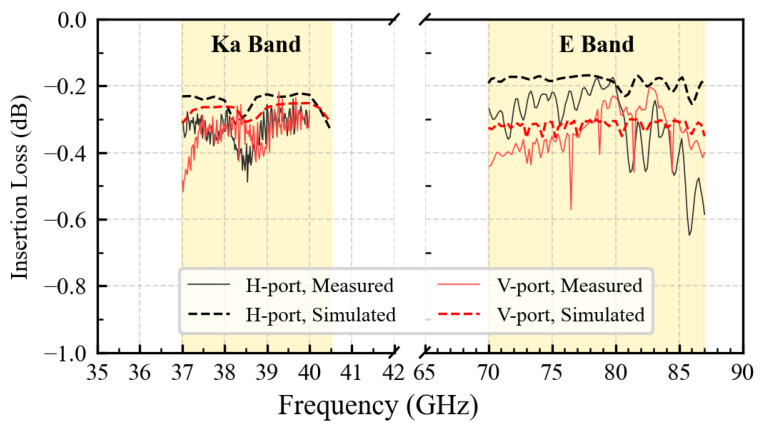
The comparison of measured and simulated insertion loss of the quad-ridged dual-band feed network for H- and V-polarization channels at the Ka- and E-band.

The extracted insertion loss is plotted in Figure 15 for both the 38 GHz and 80 GHz bands. For both H- and V-polarization channels, the insertion loss of a single feed unit remains below 0.6 dB across the two operating bands, confirming the high quality of the CNC machining and the low-loss nature of the ridge-waveguide-based design.

**Figure 16:**
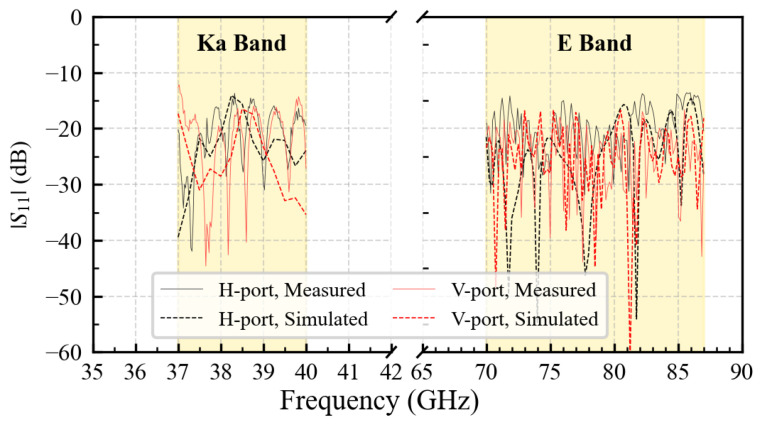
The comparison of measured and simulated reflection coefficients of the quad-ridged dual-band feed network for H- and V-polarization channels at the Ka- and E-band.

In addition to the back-to-back insertion-loss test, the input reflection coefficient at each of the four output ports and the port-to-port isolation between them were measured with the same two vector network analyzer setups (E8363B for the Ka-band and ZVA + ZVA-Z90 for the E-band), with all unused ports terminated in matched loads. These measurements characterize the impedance matching and the spectral/polarization separation provided by the proposed feed network. As shown in Figure 16, the measured $|S_{11}|$ at all four ports (H-pol and V-pol for both bands) remains below −14 dB throughout the operating bandwidths, corresponding to a VSWR below 1.5 and demonstrating excellent impedance matching at the output interfaces of the feed network.

**Figure 17:**
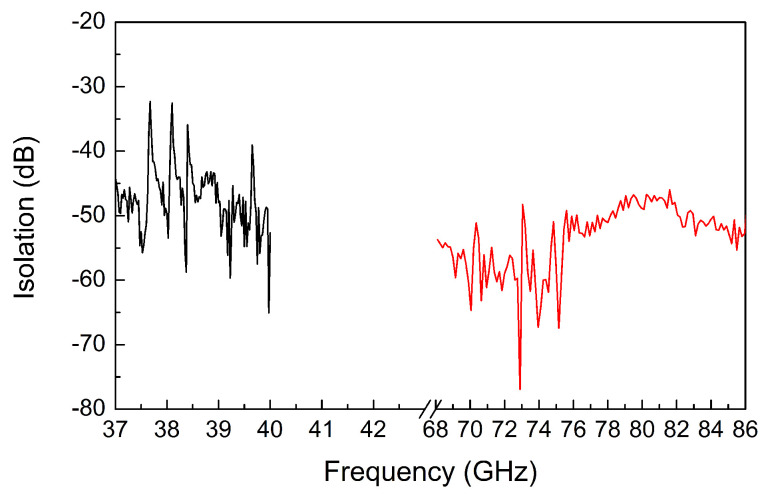
Measured intra-band isolation between the H- and V-polarized ports of the proposed feed network in the Ka- and E-bands. The black and red curves are used to distinguish the Ka- and E-band results, respectively.

The measured intra-band port-to-port isolation, presented in Figure 17, exceeds 32 dB in the Ka-band and 45 dB in the E-band, confirming the efficacy of the integrated OMT–diplexer network in suppressing cross-polarization leakage between the H- and V-polarized output ports within each band. The higher isolation at the E-band is attributed to the sharper cutoff characteristics of the rectangular waveguide in the high-pass path. Together with the back-to-back insertion-loss results above, these measurements show that the proposed feed network simultaneously achieves low insertion loss, broadband matching, and high port-to-port isolation across both 38 GHz and 80 GHz.

## 4. Conclusions

This paper has presented a compact, low-profile dual-band feed network operating at 37–40 GHz (Ka-band) and 70–86 GHz (E-band) for millimeter-wave point-to-point backhaul applications. The network integrates a quad-ridged OMT and four ridge-waveguide diplexers into a three-layer all-metal stacked architecture, eliminating the cascaded inter-stage flanges of conventional feed chains and significantly reducing the axial profile. Stepped-impedance matching stubs in the OMT junction and stepped-impedance ridge filters in the diplexers together provide broadband matching, polarization separation, and frequency multiplexing within a single compact module.

Back-to-back measurements of the fabricated prototype yield an insertion loss below 0.6 dB across both bands, confirming the high quality of the CNC machining and the low-loss nature of the ridge-waveguide-based design. The measured VSWR at the four output ports remains below 1.5 in both bands, while the port-to-port isolation exceeds 32 dB at the Ka-band and 45 dB at the E-band, demonstrating broadband impedance matching and effective spectral and polarization isolation. With its high integration level, low loss, and compatibility with standard CNC machining, the proposed network offers a practical and scalable solution for next-generation dual-band mmWave links, and the stacked architecture can be readily extended to other dual-band configurations or higher frequency ratios.

## Figures and Tables

**Table 1:** Key dimensions of the OMT structure (unit: mm).

$w_{1}$	$w_{2}$	$w_{3}$	$w_{4}$	$w_{5}$	$r_{1}$	$r_{2}$	$l_{1}$
0.8	3.2	1.33	1.2	1.04	0.54	0.914	3.1
$h_{1}$	$h_{2}$	$h_{3}$	$h_{4}$	$h_{5}$	$h_{6}$	$h_{7}$	$l_{2}$
0.845	0.9	1.4	0.64	1.19	0.995	0.62	5.0

**Table 2:** Dimensions of the 15-section stepped-impedance ridge filter (unit: mm).

n=	1	2	3	4	5	6	7	8	9	10	11	12	13	14	15
$h_{fn}$	1.10	0.94	1.10	1.10	0.92	0.75	1.07	0.90	1.12	0.70	1.07	0.72	1.05	0.59	0.90
$w_{fn}$	1.00	0.88	0.75	1.12	0.99	1.14	0.60	1.00	1.12	0.63	0.42	0.63	0.89	0.85	1.38

## Data Availability

Data are contained within the article.

## Abbreviations

The following abbreviations are used in this manuscript:

3D	Three-Dimensional
CNC	Computer Numerical Control
FSS	Frequency-Selective Surface
HPF	High-Pass Filter
LPF	Low-Pass Filter
mmWave	Millimeter-Wave
OMT	Orthomode Transducer
QRFH	Quad-Ridged Flared Horn
VSWR	Voltage Standing Wave Ratio
